# Parent engagement in co-design of clinical trials: the PARENT trial

**DOI:** 10.1186/s13063-021-05305-6

**Published:** 2021-05-17

**Authors:** Leigh M. Vanderloo, Shelley M. Vanderhout, Erika Tavares, Jonathon Maguire, Sharon Straus, Catherine S. Birken

**Affiliations:** 1 Child Health Evaluative Sciences, The Hospital for Sick Children Research Institute, Toronto, Ontario Canada; 2 School of Occupational Therapy, Health & Rehabilitation Sciences, Western University, London, Canada; 3 Genetics and Genome Biology, The Hospital for Sick Children Research Institute [Parent representative], Toronto, Canada; 4 Department of Pediatrics, St. Michael’s Hospital, Toronto, Ontario Canada; 5 Knowledge Translation Program, St. Michael’s Hospital, Toronto, Ontario Canada; 6 Department of Pediatrics, University of Toronto, Toronto, Ontario Canada

## Abstract

Evidence generated from partnering with parents to design and conduct research together may be used to refine, adjust, and modify future research approaches. This study aimed to describe the initial approaches to parent engagement in the design of the PARENT trial as well as understand parent perspectives on the acceptability and relevance of the PARENT trial and potential barriers and facilitators to participation.

Parents participating in the TARGet Kids! cohort were invited to participate in a focus group, called the PARENT panel, to co-design the PARENT trial. This focus group was conducted to capture diverse individual and collective parents’ experiences. Overall methodological approaches for the PARENT panel were informed by the CIHR Strategy for Patient Oriented Research (SPOR) guiding principles (*mutual respect*, *co-building*, *inclusiveness*, and *support*) for patient engagement in research, and facilitated through the Knowledge Translation Program in the Li Ka Shing Knowledge Institute at Unity Health Toronto. Using a Nominal Group Technique, the PARENT panel provided feedback on the feasibility, relevance, and acceptability of the proposed intervention. Findings from this work will be used to further refine, adjust, and modify the next iteration of the PARENT trial, which will also serve as an opportunity to discuss the efforts made by researchers to incorporate parent suggestions and what additional steps are required for improved patient engagement.

## Introduction

Patient-oriented research focuses on engaging patients in the research process. This type of engagement helps to ensure that research answers questions relevant to patients and uses collaboratively developed methods, and interpretation of results, with the goal of improved patient outcomes [1]. Though efforts to conduct patient-oriented clinical trials have increased [2], the degree of engagement in patient-oriented research is paradoxically poor [3] or may appear tokenistic. Patient engagement is intended to align research goals between researchers and patients, ensure research design and methods are feasible and accessible, and facilitate effective dissemination of study findings to those who contributed data as well as other stakeholders and the public. Patient engagement research has the potential to be especially impactful in trials focusing on young children, where parents can develop ongoing and genuine interactions with study teams, including clinicians and researchers, and provide meaningful input such as steering study design and dissemination of results. There is limited literature on engaging parents and young children as partners in research involving young children [4–7].

*The Applied Research Group for Kids* (*TARGet Kids!*; www.targetkids.ca) is an ongoing cohort study with embedded randomized controlled trials (RCTs), which involves parents and young children from primary care practices in three cities in Canada (Toronto, Montreal, and Kingston). TARGet kids! is a collaboration between parents, their children, child health researchers, and primary care practitioners, which aims to link early life exposures to health problems including obesity, micronutrient deficiencies, and developmental problems [8]. TARGet Kids! presents a unique opportunity to engage parents and inform research priorities, improve study recruitment and generalizability, and co-develop embedded clinical trials. One embedded randomized controlled clinical trial in TARGet Kids! is called PARENT (Parenting Addressing Early Years Intervention with Home Visits in Toronto: A Pragmatic Randomized Controlled Trial; NCT03219697), a pragmatic, parallel-group, one-to-one, superiority RCT. The aim of the PARENT trial is to determine if participation (experimental group) with a public health nurse via group parenting sessions (i.e. coaching calls addressing goal setting), and home virtual visits (i.e. addressing behaviours in the home setting) focused on nutrition, physical activity, and sleep among young children at risk of obesity can improve health outcomes such as reduced risk for overweight or obesity, and better mental health and nutrition. The control group comprised of children and parents who received usual care. In Canada, this consists of individual well-child health supervision visits, guided by the Rourke Baby Record (as endorsed by the Canadian Pediatric Society and the College of Family Physicians of Canada) [9]. Ethical approval for the PARENT trial and supporting material was received from the Hospital of Sick Children’s and the Toronto Public Health (Toronto, CANADA; REB1000054998). Informed consent was obtained from the parents of children who participated in this study.

In accordance with the International Association of Public Engagement (IAP^2^) [10], researchers in TARGet Kids! aim to inform, consult, involve, collaborate, and empower parents as co-creators. Thus, the PARENT trial was designed with the support of and in collaboration with parents of young children. Models of co-designing clinical trials are increasingly being seen as a way of addressing power imbalances by designing and delivering research in more democratic, equal, and reciprocal relationships between healthcare professionals, researchers, participants, and end-users [11]. While there have been speculations on the “best” or “correct” way of engaging in co-production and co-development and assessment of clinical trials [5], some key principles that help underpin this practice include developing peer support and engaging a range of stakeholder networks inside and outside health care; removing tightly defined boundaries among researchers, practitioners, and recipients such as parents; enabling shared control, responsibility, and mutuality; and supporting reciprocal relationships with mutual responsibilities and expectations [12]. It has been well documented that the priorities of researchers may not align well with the priorities of patients and clinicians [13–16]. Patient involvement in setting research priorities may lead to research that is more relevant to patients [17, 18]. Although patients and clinicians are increasingly recognized as important research partners, a review of priority setting studies found that only 49 of 258 (19%) studies involved both patients and clinicians in the process [19]. Nevertheless, evidence generated from partnering with parents to design and conduct research together may be used to refine, adjust, and modify future research approaches, and provide an opportunity to evaluate the process taken to engage patient partners and determine areas of strength, weakness, and need for growth.

The primary objective of this study was to describe the initial approaches to parent engagement in the design of the PARENT trial in TARGet Kids!. The secondary objectives were to understand parent perspectives on the acceptability and relevance of the PARENT trial and potential barriers and facilitators to participation, though a parent focus group.

## Methods

### Approach to patient engagement in co-design of the PARENT trial

A priority setting process was conducted to identify parent and clinician research priorities in prevention research. Using the James Lind Alliance [20] framework, this exercise revealed that the prevention of obesity and mental illness in young children were two high priority areas worthy of further investigation [21]. This was consistent with priorities of the WHO [22], Public Health Agency of Canada [23], Public Health Ontario [24], and the Canadian Task Force on Preventive Health Care [25], as rates of overweight and obesity in children under 5 years of age in Canada are currently 15% and 6%, respectively [26, 27].

Pilot work was initiated to develop an RCT to promote health behaviours and prevent obesity in young children with increased risk factors for future obesity. Existing literature was reviewed and identified using home visits was effective in trials that addressed parenting strategies for obesity prevention [28–33]. Funding for the PARENT trial was obtained through the Ontario Child Health SUPPORT Unit (OCHSU) Strategy for Patient-Oriented Research (SPOR) research network, which encouraged partnerships with patients and other patient-oriented research groups (clinicians and researchers with expertise in public health, primary care, nutrition, physical activity, mental health, early child development, obesity treatment, and epidemiology).

### PARENT panel in the TARGet Kids! cohort

Parents participating in the TARGet Kids! cohort were invited to participate in a focus group, called the PARENT panel, to co-design the PARENT trial. This focus group intended to capture diverse individual and collective parents’ experiences. Overall methodological approaches for the PARENT panel were informed by the CIHR Strategy for Patient Oriented Research (SPOR) guiding principles (*mutual respect*, *co-building*, *inclusiveness*, and *support*) [34] for patient engagement in research, and facilitated through the Knowledge Translation Program in the Li Ka Shing Knowledge Institute at Unity Health Toronto [34]. Along with the PARENT panel, the research team designed an engagement plan inspired by the IAP^2^ framework to help direct this work [35]. Using a Nominal Group Technique [36], the PARENT panel helped provide feedback on the feasibility, relevance, and acceptability of the proposed intervention. The PARENT panel prioritized research outcomes in the PARENT trial and contributed to the list of potential child and family barriers and facilitators to participating in the intervention. Through informing how to tailor both TARGet Kids! and the PARENT trial to increase participation, parents had the opportunity to *co-build* the PARENT trial research to meet their interests and needs.

### Participant recruitment for the PARENT panel

Recruitment advertisements were posted in TARGet Kids! primary care practices and on the TARGet Kids! website as well as sent via email to TARGet Kids! participants. An online survey was used to determine eligibility (must be involved in TARGet Kids!, have a child between age 12 months and 6 years, have a computer and internet access) and gain informed consent. Background information about the PARENT trial was shared via email to interested and eligible participants prior to the focus group to enable them to contribute to the discussion. Participants received a $15 gift card for local grocery store for participating.

### Data collection and analysis

Once informed consent was obtained, a 60-min focus group was conducted using WebEx, an online web conference platform, which enabled participants to review a brief video presentation about the PARENT trial prior to the focus group discussion. PARENT panel participants were engaged in meaningful discussions through open-ended questions facilitated by a trained qualitative moderator, using a 10-item semi-structured interview guide created by the Knowledge Translation Program (in consultation with the PARENT research group; Appendix). The focus group discussion was digitally recorded and transcribed verbatim by a member of the Knowledge Translation Program and member-checked by participants prior to analysis.

Data analysis was informed by the Theoretical Domains Framework (TDF; average silhouette value 0.29, good face validity) [37] to determine root causes of barriers and facilitators to engagement in the PARENT trial. The TDF involves coding interview transcripts into theoretical domains (*deductive analysis*) and is well-suited for explaining implementation problems and informing implementation interventions [37]. Data were analysed by generating overarching themes from the selected theoretical domains (*thematic inductive analysis*). The overarching themes represent the factors which were perceived to influence performance of the target behaviour. Once identified, themes were reviewed by the study team to develop and apply changes to the PARENT trial protocol to ensure parent views and suggestions were incorporated. The SPOR Patient Engagement Framework [34] was also used to align and support the synthesis of participants’ recommendations for increasing engagement in the PARENT trial.

## Results

In 2019, a total of seven parents (mean age = 31.4 [6.2] years, 100% female, 71% European descent, 86% employed full-time) were recruited for participation in the focus group. As evidenced by supporting quotations regarding trial perceptions, the PARENT Trial was well-received by participants (Table 1). Overarching themes related to increasing parent engagement in the PARENT trial (i.e. barriers and facilitators) emerged from the focus group transcript (Table 2) and are described below according to different aspects of the study.

**Table 1:** Parent perceptions of the PARENT trial

Perception	Example quote	Positive or negative?
Parents perceive the PARENT trial positively, offering many potential benefits around addressing childhood overweight and obesity	“my overall thoughts about the, what I read about the Parent Trial and saw is that it’s an excellent study and it’s very interesting to see how you’re looking to collect data or how you’re collecting data and what you’re doing with that, but it looks like it’s a really, it should hopefully be a very effective study (anyway).”	Positive
	“… so the things that I like about the study, obviously, are: the objectives for the study (around childhood obesity) but also being able to go through all of the questions – it really sparks ideas (for me personally) around what I should be doing as a first-time mom, so for example: there are a lot of questions in that questionnaire around screen time and so that obviously highlighted something that is related to childhood obesity for me.”	
	“When I read through the materials (and also watching this presentation) I thought it was really cool – I think it’s a really interesting way to, maybe, engage some parents that don’t know what they can do about childhood obesity other than eating healthy, and even when you say eating healthy I feel some parents (and even including myself), like “what does that really mean? What sort of food am I going to cook and feed my kids?” and then, of course, if you come from different backgrounds with diverse foods “how do I work that into my own culture?” I think having assistance and, maybe, someone keeping you accountable (too) is really interesting, so I don’t know if that’s the program running forward or if you’re just testing it out, but I think that’s, keeping people accountable is also really useful and good to do.”	
	“of course home visits, accountability, getting the extra support that you could get as a parent to improve your child’s health, all of that…”	
	“I just wanted to add that in terms of what they might see as benefits: things like the, just getting assistance (like for the home visits) – if they have questions that they want to ask – or like the group sessions just learning things that they might not otherwise know.”	
Home visits are especially beneficial as they encourage accountability and offer assistance	“I just wanted to add that in terms of what they might see as benefits: things like the, just getting assistance (like for the home visits)…”	Negative
	“I thought, first of all the home visits was a cool idea”	
	“Yeah, home visits really keep people accountable and I think giving them a person and a face to talk to or direct their questions to is really great because that engages them in their journey to being healthier or preventing obesity and what not.”	
Alternative options may more appropriately assess outcomes related to overweight and obesity than BMI	“I feel like they’re quite appropriate, except for maybe the BMI because I feel like I’ve read a lot of criticism against that recently (maybe over the past year or 2) and I know it’s still the standard, but shouldn’t we be exploring different ways to measure outcomes?”	Positive
	“I guess BMI also stuck out to me and I just kind of wonder if it’s, when you hear it’s problematic if it’s more for adults than it is for children ‘cause I think it has to do with muscle mass and maybe that’s not the same factor with children, but maybe a measure that could be used is body fat percentage rather than BMI – so, that’s just something that come to mind.”	
	“if we’re talking about obesity and then trying to prevent it from happening or controlling obesity, would physical activity, like hours of physical activity or decrease in screen time or something else that is more than just weight and fat? I only say that because I know, and I’m also not a medical professional so correct me if I’m wrong, but I also know a bunch of people who are bigger and are considered obese but they’re so healthy and they exercise a lot and they eat really, really healthy so I don’t know if there are genetic factors (or something else) that contribute to something and that we could take in to consideration – ‘cause I almost feel BMI is unfair for those people.”

**Table 2:** Elicited barriers and facilitators from participants regarding the PARENT trial

Practice	Barrier or facilitator	Example quote	Theoretical domain
Blood samples	*Barrier* – Parents identify the blood samples as a factor that could harm their child	“I guess for potential harms I’m trying to remember if I said ‘no’ to the blood sample or if I just haven’t been ask yet – it’s been hard to weed out different studies I’ve been asked to participate in – but I know I’ve said ‘no’ to that before so I see that as a potential harm”	**Belief about consequences**: “Acceptance of the truth, reality, or validity about outcomes of a behavior in a given situation”
		“I think hearing more about the blood sample might also just be ‘first-time mom’ worries but just I wasn’t sure if it would be done at XXX [hospital name – 00:44:28] or by my family doctor – like “would it be by someone who’s taken blood from a little baby before?” Like she’s 2 now but when I first started, she was 6 weeks old and I was like “what? No way”	
	*Barrier* – The blood sample may elicit negative emotions, including nerves and fear	“The things that I don’t like [laughing] just being honest) I’m a little bit nervous about the blood sample that’ll be taken, but that’s as a first-time mom – just scared about that for my little one.”	**Emotion**: “A complex reaction pattern, involving experiential, behavioural, and physiological elements, by which the individual attempts to deal with a personally significant matter or event”
	*Barrier* – Providing blood sample without receiving potential trial benefits can strengthen perception of harm	“…in the control group it just feels a little bit extra like “ugh, so we got to give the blood but we don’t get the at-home visits or the one-on-one support” so I guess that’s what felt like a potential harm for me.”	**Reinforcement**: “Increasing the probability of a response by arranging a depending relationship, or contingency, between the response and a given stimulus”
Home visits	*Barrier* – Home visits may be threatening due to concerns about judgement	“In terms of not like: I can see people being concerned about the home visits – about “what are they looking for? Are they going to judge the state of my home?”, you know, just things like that that people I know can be concerned about when people are coming into their homes”	**Belief about consequences**: “Acceptance of the truth, reality, or validity about outcomes of a behavior in a given situation”
		“I would also have concerns if there were home visits – I think my worry would be “ugh, what are they looking at? Are they going to be looking at my sink dishes in it, or are they judging the whole atmosphere here, or are they just sitting to have the talk?” so hearing what to expect.”	
		“I would say mostly just a misunder--------, like not understanding what the home visits are would probably be a big barrier. Like I said, people thinking that you’re coming in judging them as opposed to coming in to assess.”	
	*Barrier –* Public health nurse approach may not be congruent with the nature of support parents want to receive in the home visits	“I find that public health nurses are really good at giving you a summary of what the recommended guidelines are but I find that public health nurses can sometimes be rigid and so if you’re like “oh my gosh, my kids throw a tantrum every time they demand juice” I feel like public health nurses will often say “oh, well, it’s best not to give juice – give water””	**Social or professional role and identity**: “A coherent set of behaviours and displayed personal qualities of an individual in a social or work setting”
	*Facilitator* - Some parents perceive expertise and approach of a public health nurse to be appropriate for their needs	“I like having a public nurse there. I feel like he or she would be quite knowledgeable about, well, they would’ve seen many children and many different types of patients so they have experience as to, maybe, how kids might react to less screen time (if they have any strategies on encouraging that), so I think the nurse is really great”	**Social or professional role and identity**: “A coherent set of behaviours and displayed personal qualities of an individual in a social or work setting”
Home visits and blood samples	*Barrier* - A lack of knowledge around the purpose and procedure for different trial activities may deter participation	“Maybe hearing about how other little babies react to getting their blood checked that would help me get information about that; and, yeah, I guess I would also have concerns if there were home visits.”	**Knowledge**: “An awareness of the existence of something”
		“ugh, what are they looking at? Are they going to be looking at my sink dishes in it, or are they judging the whole atmosphere here, or are they just sitting to have the talk?” so hearing what to expect.”	
		“I think the only thing that’s stood out for me is: reading in the protocol (or the consent) that it’s specifically for kids who are at a high-risk for childhood obesity, and I think when I read that I was like “what? My kid’s at high-risk for obesity? I didn’t even know that” so just some questions that come up with recruitment around that.”	
		“I don’t know where they got the information that my child was at risk for childhood obesity – [laughing] I don’t know if it’s based on my health background or the health file or something – so just hearing from the Research Assistants why we’ve been chosen.”	
	*Barrier -* Scheduling and time constraints may impact ability to participate in group sessions and home visits	“My thought on the group sessions though, I myself have difficulty attending that so I imagine that would be difficult for other families as well.”	**Environmental context and resources**: “Any circumstance of a person's situation or environment that discourages or encourages the development of skills and abilities, independence, social competence, and adaptive behaviour”
		“Yes, and they’re weekly, and, so I’m not sure, of course it’s a group and there has to be a consensus for the time. It’s going to be a little bit of time management every single time you have to be a part of that group.”	
		“Just because I know it’s difficult to schedule your time around attending a session in person.”	
		“… from a time perspective I personally am involved with home visits from a XXX [city name – 01:07:03] health public nurse right now and someone who comes to our house regularly (like every 2-3 weeks or so) and I’ve been part of the program for the past year and at this point I’m ready to end it – I want that time back [laughing] – so I would just say ‘time’.”	
		“Yeah, I just think the, they become a bit repetitive after a while, and it’s only about an hour and it’s in the home, so I just found that they work your schedule but just wanting that time back – especially if it falls through nap hour.”	
Recruitment	*Barrier -* Parents may have difficulty distinguishing PARENT Trial study from overload of similar information	“No, I think what’s going through my mind now is, I find it hard, I think because they’re all through my Doctor’s Office and there’s lots of different projects going on that they’re hard for me to parse out what’s what [laughing]”	**Memory, attention, and decision processes**: “The ability to retain information, focus selectively on aspects of the environment and choose between two or more alternatives”
	*Facilitator* - Leveraging pre-existing participant points of contact may facilitate recruitment	“in one way I kind of like that because I find that the studies are so not intrusive that I barely notice them – like I think so far it’s just been the long questionnaires and measuring – and so the recruitment has been just so easy that I haven’t especially noticed it so I think it’s just getting emails from things that I participate in through my Doctor, so it’s been easy.”	**Memory, attention, and decision processes**: “The ability to retain information, focus selectively on aspects of the environment and choose between two or more alternatives”
			**Social or professional role and identity**: “A coherent set of behaviours and displayed personal qualities of an individual in a social or work setting”

### Target condition

Participants felt the trial would be effective in attenuating childhood overweight and obesity. They believed the material provided was detailed, evidence-informed, and actionable (i.e. provided many useful tips). Home visits were highlighted as being especially beneficial as they encourage accountability and offer assistance. Participants felt that visits from public health nurse would provide a unique opportunity to ask questions, receive support, and add accountability to the proposed behavioural changes. The primary reservation noted by participants was the use of BMI to assess weight-related outcomes in children. Participants questioned its utility in the study and were less convinced that it was the most appropriate measure to evaluate outcomes of overweight and obesity in children.

#### Foster opportunities for peer support (TDF domain: “social or professional role and identity” and “emotion”)

Parents were interested in engaging with and learning from other parents who were going through similar experiences with their children (e.g. developing an online platform to share experiences). They believed that the PARENT trial had great potential to enhance group connectedness if given the opportunity to interact with other participants’ parents: “...it’d be interesting to have that social engagement with the parents as well, and have parents interact with each other (maybe swap tips and stuff like that). I don’t know if that’s possible, but it would make it more interesting” [P2].

#### Memory, attention, and decision processes

Participants felt it may be difficult for parents to differentiate between the different trials or studies being offered through their primary care physician’s office and at times felt overloaded with the amount of similar information being shared. However, participants also noted that because the request to participate was coming from their healthcare clinic (wherein they already a trusted and established relationship), they may be more likely to partake in the trial.

### Trial design

Focus group respondents provided detailed recommendations for improving the *recruitment*, *engagement*, and *intervention* of participants in the PARENT trial (Table 3).

**Table 3:** Parent-reported PARENT trial recommendations

Practice	Recommendation	Example(s)	Type of recommendation
**Home visits**	Parents had suggestions for the role and expertise they would like to be present in the individual leading the home visits, which included a focus on behavioural strategies	“I feel that maybe someone who works with kids a lot – I don’t know if that would be a Psychologist or something – that would have strategies too that they could offer, that could help you encourage kids to do certain things because once they get in to the habit of, for example: drinking a lot of juice like “how do you then wean them off that to water (which is completely different than juice)” – right? Or if they are getting a lot of screen time “how do you wean them off of screen time so they’re not angry and screaming and basically causing hell in your home?” so, I think someone maybe like a Psychologist that can give you some tips would be very helpful – especially if you’re trying to implement them in a very short period of time (like 6-months).”	Protocol recommendation. *Related SPOR domain* – **Co-building**: “Patients, researchers and practitioners work together from the beginning to identify problems and gaps, set priorities for research and work together to produce and implement solutions.”
		“yeah, stuff around “the best ways to feed them nutritionally but also keep them interested in food”, and just making sure that you’re aware what, where your kid is supposed to be at in terms physical activity (that kind of thing) because although we research that sometimes	
		we’re not sure if it’s, if the information we’re finding is correct (you know, just things like that) and then maybe with toddlers things around “how to deal with behaviour [like temper tantrums and stuff like that]” – like I go to programs on a regular basis where I’m learning how to deal with that but not everyone has those resources or the time or the abilities to do that as well.”	
		“so, I would like to get some help with time management with the toddlers when it comes to their health. If a kid is going to school or if you’re working or you’re going back to work fulltime there’s so little time you’re going to be spending with your child – it’s not going to be more than 2-3 hours every day – so you have to make sure that whatever you’re feeding them is healthy or to make sure there’s, just about enough screen time and not more than, enough physical activity and everything has to be done in those 2-3 hours; so, that is something I would like a public health nurse to help me out with – like how to manage that time wisely.”	
		“in terms of physical activities, I think it would be really great to get some ideas for physical activity for this weather – like indoor physical activities, other than jumping around in the house. I think that’s just something I’m lost just in terms of ideas and how to keep them engaged in a physical manner, so that is something I need help with – some ideas.”	
		“a public health nurse I feel like he or she would, can give you medical advice or “how much, how many servings of fruit to serve? [or something like that]” but when it comes to time management, I feel like it’s a, maybe, councilor or something else.”	
		“what type of professional would be helpful with the visits? I don’t know if this makes sense but I almost feel like someone who can do a harm reduction approach to healthy eating with your child’ and I find that public health nurses are really good at giving you a summary of what the recommended guidelines are but I find that public health nurses can sometimes be rigid and so if you’re like “oh my gosh, my kids throw a tantrum every time they demand juice” I feel like public health nurses will often say “oh, well, it’s best not to give juice – give water” but I would love to have someone who, like an ECE person or someone who’s more familiar with child behaviour to be like “oh, you know what a useful tip is? Give it 75% of water and a splash of juice and that makes them happy and it’s more healthy” like kind of giving, I don’t know, daily tricks rather than guidelines.”	
		“when you say ECE are you speaking of an ‘early childhood educator’? P6: That is what I’m speaking of but I’m, not necessarily that it has to be that – I was just thinking of people who are, have a child-specific educational training background.”	
**Recruitment**	Strategies should be employed to ensure recruitment materials effectively engage the attention of potential participants	“I: No, no, that’s totally fine and I think that comment’s still very valuable if you think that email can sometimes get lost in your inbox and you would prefer something like a hard mail copy or something directly in the Physician’s Office it’s a very valuable comment.	Protocol recommendation.
		P5: “Yes, yes – that’s what I tried to get at (it just didn’t jump out or it didn’t stand out when I first received it) so something a little more direct I think would’ve been a better way to do that.”	
**Home visits**	Information about the nature and objective of different potentially invasive interactions between participants and researchers should be clearly outlined	P4: “I guess just a general, like you’re looking at, again, the type of toys the child has – not the quality of the toys. You know, just what kinds of things would facilitate, you’re not necessarily looking at, that they haven’t cleaned behind their fridge recently [laughing] – you know, that kind of thing.”	Engagement recommendation. *Related SPOR domain* – **Support**: “Adequate support and flexibility are provided to patient participants to ensure that they can contribute fully to discussions and decisions. This implies creating safe environments that promote honest interactions, cultural competence, training, and education. Support also implies financial compensation for their involvement.” AND **Mutual respect**: “Researchers, practitioners and patients acknowledge and value each other’s expertise and experiential knowledge.”
		“so just hearing what the actual process would look like and maybe hearing about how other little babies react to getting their blood checked that would help me get information about that…”	
**Group sessions and outcome measures**	The appropriate structure and support must be in place to ensure parents can attend and complete all aspects of the PARENT Trial	“I know it’s, those group sessions are in-person but would it be possible to have something that is online – that is, maybe, similar to this – or have an online lecture (or whatever) as an option? Just because I know it’s difficult to schedule your time around attending a session in person.”	Engagement recommendation. *Related SPOR domain*– **Support**: “Adequate support and flexibility are provided to patient participants to ensure that they can contribute fully to discussions and decisions. This implies creating safe environments that promote honest interactions, cultural competence, training, and education. Support also implies financial compensation for their involvement.”
		“maybe to have something recorded and the person can watch whenever time is convenient but it needs an opportunity to ask questions at some point (right?) – like an online course so the person can adjust, like Netflix, can see whenever you have a set time – but I think this one-to-one is important to be able to have our feedback.”	
		“I just wanted to add, for me personally, it would be, if there was any childcare option then I could do it pretty much any time.”	
		“From personal experience weekends would be easiest ‘cause that’s when childcare is more available to us [laughing].”	
		“P3: XXX [name – 01:17:33] here: I don’t think, I would not mind if I had to do a diary of physical activity for my kid (or whatnot) or for myself – I just feel that as long as you make it easy so I don’t have to recall what I did 2 weeks ago (‘cause I don’t remember) I would be happy to do anything as long as it was easy.	
		I: Thank you; and do you mind just elaborating on, specifically, what would make it easy? So, you mentioned having the questionnaires in close proximity to the behaviour – is there anything else that would make it easier for you?	
		P3: If it were online it would be very easy as well [laughing].”	
**PARENT trial overall**	Foster opportunities for peer support	“then just to further one of the comments already made about providing feedback if it’s online: maybe this could be a consideration, I remember when I was taking some University courses we would have, in our class it would be our group and then we could interact with each other through a platform and the teacher (or whatever) would post up the assignments or some of the lectures or some questions and then all of us could answer (like a forum) – not sure if that’s something that can be considered but it’d be interesting to have that social engagement with the parents as well, and have parents interact with each other (maybe swap tips and stuff like that). I don’t know if that’s possible, but it would make it more interesting.”	Engagement recommendation *Related SPOR domain* – **Support**: “Adequate support and flexibility are provided to patient participants to ensure that they can contribute fully to discussions and decisions. This implies creating safe environments that promote honest interactions, cultural competence, training, and education. Support also implies financial compensation for their involvement.”

#### Use alternate communication methods for participant recruitment (to distinguish between different trials within the TARGet Kids! cohort; TDF domain: “environmental context and resources”)

Parents reported that trial recruitment materials were not easy to differentiate from other emails related to the ongoing cohort study. Suggestions for methods that may be more effective in gaining attention, as stated by one participant: “... email can sometimes get lost in my inbox, so I would prefer something like a hard mail copy or something directly in the Physician’s Office...” [P5].

#### Share detailed information about potentially invasive trial activities (TDF domain: “Beliefs about the consequences”, “knowledge”, and “reinforcement”

Parents recommended sharing detailed information about the purpose of, and protocol for, potentially invasive components of the trial. For example, many parents had concerns regarding their young children undergoing blood tests related to obesity outcomes, as expressed by “The things that I don’t like [laughing] just being honest, I’m a little bit nervous about the blood sample that’ll be taken, but that’s as a first-time mom – just scared about that for my little one” [P3] and “…in the control group it just feels a little bit extra like “ugh, so we got to give the blood but we don’t get the at-home visits or the one-on-one support” so I guess that’s what felt like a potential harm for me” [P5]. Home visits by public health nurses were also noted as a friction point: “In terms of not like: I can see people being concerned about the home visits – about “what are they looking for? Are they going to judge the state of my home?, you know, just things like that that people I know can be concerned about when people are coming into their homes” [P1] and “I would also have concerns if there were home visits – I think my worry would be ugh, what are they looking at? Are they going to be looking at my sink dishes in it, or are they judging the whole atmosphere here, or are they just sitting to have the talk? So hearing what to expect” [P3].

#### Consider professional role in the discussion of sensitive information (TDF domain: “social or professional role and identity” and “memory, attention, and decisions processes”)

Parents identified that as a participant in the PARENT trial, they would want to receive information about *why* they were being recruited to participate and to be able to discuss any related medical concerns with their physician. One parent noted “I think the only thing that’s stood out for me is: reading in the protocol (or the consent) that it’s specifically for kids who are at a high-risk for childhood obesity, and I think when I read that I was like “what? My kid’s at high-risk for obesity? I didn’t even know that” so just some questions that come up with recruitment around that” [P4].

### Intervention

#### Belief about consequences

Panel participants were apprehensive about having their young children undergo unnecessary blood work. They were nervous about the undue pain and discomfort this might cause for their children.

#### Emotion

Due to the young age of the children, parents expressed negative feelings about their kin providing a blood sample and elicited negative emotions, including nerves and fear.

#### Reinforcement

Parent panellists expressed that those assigned to the control group may be less likely to have their child provide a blood sample as they will not be receiving anticipated trial benefits allotted to those in the experimental group (e.g. home visits, support, group sessions, information) and thus reinforce their perception of harm.

#### Social or professional role and identity

‘*Who*’ was delivering the intervention was raised as an important point. While some felt that the approach of public health nurses may not be congruent with the nature of support parents want to receive in the home visits (and that a social worker or nutritionist may be better), whereas others perceived the expertise and approach of public health nurses to be appropriate for their needs.

#### Knowledge

Panel participants noted a lack of understanding regarding what the public health nurses were looking for during their home visits and expressed an uneasiness about feeling judged in their homes. As well, others felt that a lack of knowledge around the purpose and procedure for different trial activities, including bloodwork, may deter participation.

#### Environmental context and resources

Due to the various and diverse schedules of families, panellists felt that scheduling and time constraints may impact the ability of participants to join in group sessions and home visits.

#### Provide support to engage in the PARENT trial (TDF domain: “environment context and resources”)

To mitigate scheduling constraints for the group sessions, parents suggested offering part of the sessions through an online platform that could be accessed at their convenience, as mentioned by one parent: “maybe to have something recorded and the person can watch whenever time is convenient but it needs an opportunity to ask questions at some point (right?) – like an online course so the person can adjust, like Netflix, can see whenever you have a set time –...”. Parent participants also highlighted that availability of childcare would be very helpful at the sessions.

### Application of findings

Many changes were made to the PARENT trial protocol based on the feedback of parents prior to the launch of the trial and as a way of circumventing identified barriers. Specific modifications—organized by *recruitment*, *engagement*, and *intervention—*are presented in Table 4. Overall, from a *recruitment* perspective, it was expressed that developing meaningful and authentic connections between parent participants and researchers was key to establishing a solid rapport. Providing support for parents—whether in the form of embracing various methods of involvement (virtual or in-person), reducing the number of sessions, and ensuring all forms of communications were lay friendly—were highlighted as important points for consideration moving forward. In terms of *engagement*, ensuring the objectives of the study were clear and that the findings were accessible and widely distributed were highlighted as important contributors to a successful trial from a participant engagement perspective. For the *intervention* component, ensuring that participating parents felt their involvement and contributions was being taken seriously and integrated where possible was highlighted during panel discussions. Examples include modifying research approaches (where possible/appropriate) and touching base with participating families throughout the trial.

**Table 4:** Resulting modifications to trial and application of findings

Category	Modification
Recruitment	• Ensuring all participants have effective ways of participating in decision-making structures and feeling that they have a real say in them. This will, in part, be achieved through semi-annual PACT meetings.
	• Engaging in good practices in regarding health literacy (speaking slowly, avoiding jargon and acronyms, repeating points to improve comprehension, encourage and expect all patients to ask questions, check understanding and recall).
	• While the research team has encountered difficulties in developing an online system for parent participants to interact due to privacy concerns, we have raised the idea of participants sharing emails or engaging in a Facebook group if preferred.
	• Embracing different forms and methods of involvement. In addition to in-person contact, consider the possibility of implementing telephone or online discussions with participating families to collect data. Utilizing a variety of media to interact with families will help address barriers related to time constraints and lack of childcare provision.
	• Collaborating with the Early Childhood Education program at a local college to offer free childcare (to increase accessibility of trial to participants).
	• Reducing the number of sessions with public health nurse from 10 to 8 (to accommodate parent’s busy schedules).
	• Providing the option for parents to join the sessions via Zoom, videoconference (to accommodate parent’s busy schedules)
Engagement	• Developing new recruitment material using various media to promote the study and provide important information about the trial.
	◦ A whiteboard video that can be emailed to eligible participants at recruitment.
	◦ A revised recruitment poster that can be displayed in medical offices.
	◦ Using different coloured paper to help participants differentiate between different studies they may be participating in within the TARGet Kids! cohort
	• Providing further explanation to potential participants regarding the purpose of the PARENT trial, and revising plans to better communicate findings from this work with participants promptly and in a more accessible manner.
Intervention	• Providing an option to use online conference calling for group sessions, providing childcare during sessions
	• Re-formatting the questionnaires to assist in completion
	• Ensuring that participating parents feel their involvement and contributions is being taken seriously and integrated where possible. This will include taking findings from the focus group, modifying research approaches (where possible/appropriate), and touching base with participating families throughout the trial.
	• Developing a feedback page to report results of baseline findings to be provided to the family and reviewed at the home visit with the public health nurse.
	• Developed an integrated curriculum with existing evidence-informed program, Chicago parent program, for the intervention program.
	• Added a follow-up visit at 12 months (to evaluate effectiveness of intervention in the longer term; to determine if changed in outcome measures are different, same with the baseline and 6-month follow-up).
	• Added coaching calls (to improve engagement and rapport between public health nurse and participant).

## Discussion and future directions

The present study provides an example of the methodological approach and preliminary engagement of parent participants as co-designers in cohort embedded RCTs in young children. Results of this study highlight the importance of gaining of parents’ feedback when designing a clinical trial in early childhood. As parents contribute valuable time to participate in the TARGet Kids! cohort study, ensuring their ideas, concerns, and priorities are included in planning and implementation is important to conducting clinical research which is most relevant for children and families. The familiarity of parents with the processes, recruitment and communication techniques of TARGet Kids!, as well as possessing good rapport with the TARGet Kids! research staff, enabled the successful involvement of parents for this study.

Developing stronger patient involvement in the organization and delivery of clinical trials is central to health service and research reform [38]; panellists in this study highlighted the importance of parents serving as co-builders in the trial and to provide suggestion on the role and expertise of individuals involved in leading and/or delivering intervention components (particularly, the home visits). This recognition reflects evidence that parents of young patients can be involved and make a difference at multiple stages of research planning and delivery [39]. In accordance with Ocloo and Matthews’ narrative review on patient and public involvement in healthcare [12], the barriers elicited from the PARENT panel fall primarily under “communication issues” (i.e. access to and understanding of information and understanding of the study), “tokenism” (i.e. symbolic effort to be inclusive to members of a particular group in order to give the appearance of equality), “poor health literacy” (i.e. difficulty understanding the trial and information required to participate), and “inadequate information about involvement” (i.e. unsure about how to contribute and whether contributions will be used/applied).

Our findings highlight the contribution of patient-oriented research from the initial stages of designing and implementing a clinical trial. In line with published work by INVOLVE (UK) [7], parent input can assist researchers with constructing and conducting trials from a logistics and process perspective (e.g. enhanced recruitment and participant buy-in, improved compliancy and study connection, and stronger retention) to ensure relevant research questions are asked. Patient engagement in studies conducted among other groups has resulted in higher recruitment and retention rates [40], better acceptability of treatment options [12], relevance of research findings [2], and improved translation of research findings into clinical practice [41]. However, long-term evaluation of patient engagement in research is not yet available, and limitations may exist such as lack of available resources to meaningfully engage patients in all aspects of research, including training, compensation, and time [12]. Patients taking part in research have highlighted the need to clearly define roles based on unique skills or expertise each patient brings to the team, and provide feedback and updates about their impact on research conduct and outcomes [42]. This has been suggested to improve the perceived value of participation to patient partners, which is key to sustained, trusting relationships between patients and researchers [42]. While this study focused on consulting parent partners for input during the planning phase of the PARENT trial, it is important to consider the spectrum of patient engagement (inform, consult, involve, collaborate and empower) [10] and continue to extend opportunities for parent participation throughout the study where possible.

Learning from this experience, we have established a Parent and Clinician Team (PACT) within TARGet Kids! to involve parents in all stages of co-design for all cohort embedded clinical trials. Both structured and unstructured discussion during semi-annual meetings allows for collaborative relationships to be built and provides space for parents to bring concerns, questions and ideas from their communities to the TARGet Kids! team. Between meetings, parents have the option to volunteer as partners for specific studies, which includes reviewing and providing feedback on grant proposals, study protocols, and procedures. On an ongoing basis, parents make recommendations for knowledge translation and dissemination strategies that are accessible and impactful to their families and communities, such as print materials, social media and email newsletters. Based on the findings from the focus group, it is believed that this approach will continue to offer support (peer or expert) and create a sense mutual respect and reciprocity between clinicians, researchers, and parent partners.

Limitations of this study include the small sample composed entirely of mothers and primarily of high socioeconomic status and European descent, thus constraining the generalizability of these findings. Additional focus group discussions may have resulted in more fruitful discussions and a wider breadth of responses. However, given that the goal of these discussions with parents was not to reach saturation, but to gain a preliminary view of the acceptability and relevance of the PARENT trial and potential barriers and facilitators to participation, the voices and opinions of seven participants were deemed sufficient to achieve the study’s goals. Because of the tight timeframe between collecting and applying participant suggestions and deploying the trial, recruiting parents and coordinating a meeting time that worked for all invitees proved challenging. Consequently, it was important for the research team to remain flexible and discuss novel ways to bring the participants together, hence the use of a virtual interviewing platform during the evening.

With the PARENT trial now underway, evaluations are being recorded to ascertain whether the suggested changes have resulted in better study logistics and improved outcomes compared to previous recruitment efforts undertaken by the TARGet Kids! research team. A second round of focus groups will take place with parent participants in the PARENT trial to elicit their feedback on their overall perspectives on the delivered protocol and recruitment methods, to gain perspectives on actual barriers/facilitators to parents’ participation, and to identify suggestions for improving parent engagement. The findings from this work will be used to further refine, adjust, and modify the next iteration of the PARENT trial, which will also serve as an opportunity to discuss the efforts made by researchers to incorporate parent suggestions and what additional steps are required for improved patient engagement.

## Conclusions

The PARENT trial provided an opportunity to engage parents around an obesity prevention clinical trial embedded in a cohort study. A focus group with parents prior to the trial launch provided important insights which enhanced the study design. We hypothesize that parent engagement in the design of the PARENT trial will improve the relevance, feasibility and impact of the trial results.

## Data Availability

Available upon request.

## Appendix

### Focus group interview guide

#### Intro/warm-up questions

1. Overweight and obesity in young children is increasing in Canada. What are your impressions about this?

- *Probes: Is this something that is relevant to you? Is this something you think about in relation to your child? Has anyone in your family struggled with weight issues? How would you describe your awareness of this issue? What do you pay attention to in the news about overweight and obesity in young children? Is addressing overweight and obesity in young children important to you?*

#### TARGet Kids! questions

2. Thinking back to when you were first asked to be a part of TARGet Kids!... [*Interviewer PAUSES; let parents think about it]…* Can you describe how that process went?

- *Probes: Were you clear about what you were being invited to participate in? Were you clear about what was being asked of you? Can you describe how you made your decision to participate in TARGet Kids!? What were your reasons for joining TARGet Kids? Is there something about the first contact or invitation that could have been improved upon to help with your decision making process?*

3. How has your experience been since joining TARGet Kids?

- *Probes: How would you describe your involvement with TARGet Kids? What is your experience like as a parent participating in the clinical pieces? What is your experience like participating in the research activities (e.g., interacting with the TARGet Kids! research assistants)? What is the experience like for your child? What inspires you to continue being involved with TARGetKids?*

4. What are some suggestions for improving parent engagement in TARGet Kids!?

- *Probes: How would you suggest engaging parents to get them involved in research studies like TARGetKids!? How would you make sure that parents stay engaged? If you were recommending the study to another parent, what reasons would you give?*

### *Show PARENT trial slide show or white board video*

#### PARENT trial questions

5. Thinking about the proposed PARENT trial and introduction slides that you were presented with, what are your overall thoughts about the proposed study?

- *Probes: What do you think families might like? What do you think families might not like? What do you see as benefits? What do you see as harms? Is there anything you are not sure about?*

6. What are your thoughts on the design of the proposed study?

- *Probes: How do you feel about having an intervention group vs. control group? What do you think about the 10 weekly group sessions on health behaviours? What do you think about the parenting support with home visits? Who do you think would be the most ideal person to deliver the parenting support sessions and home visits (e.g., Research assistant? Public Health Nurse? Dietitian? Social Worker? Parents who participated)? What kind of expertise would you want in them? How do you feel about the duration of the study (i.e., 6 months)?*

7. What do you believe to be factors (i.e., barriers and facilitators) that influence participation in the proposed study?

- *Probes: What do you think are the barriers to participating in the group sessions? What do you think are the facilitators to participating in the group sessions? What do you think are the barriers to receiving parenting support through home visits? What do you think are the facilitators to receiving parenting support through home visits?*

8. How do you feel about the methods used to recruit parents to participate in the study?

- *Probes: Are the recruitment methods clear? What do you think about the video to invite parents into the study? What are your suggestions for improving recruitment efforts? What are your suggestions for recruiting in other ways?*

9. What are your thoughts on the outcomes of the proposed study? (i.e., weight/height (BMI), mental health).

- *Probes: How do you feel about these outcomes and their appropriateness for this trial? Would you suggest other outcomes? What are your thoughts on having to measure extra things outside of TARGet Kids! (e.g., the Parenting Scale, Cost Questionnaire, and Depression, Anxiety, and Stress Scale, your child having to do blood work 6 months earlier than usual)?*

10. Is there anything else you would like to add?

Thank you for sharing your insights with me. If there is nothing further anyone would like to add, I’d like to take a moment to inform you about the feedback survey. [TARGet Kids! team member] will be emailing you a short 15-minute survey regarding your experience with this Parents’ Panel to complete.

Thank you again for your participation.
